# ﻿Revision of the family Haliplidae (Insecta, Coleoptera) in Japan

**DOI:** 10.3897/zookeys.1168.99302

**Published:** 2023-07-03

**Authors:** Masakazu Hayashi, Tomofumi Iwata, Hiroyuki Yoshitomi

**Affiliations:** 1 Hoshizaki Green Foundation, Sono, Izumo, 691-0076, Japan Hoshizaki Green Foundation Izumo Japan; 2 Toyama Science Museum, 1-8-31 Nishinakano-machi, Toyama, 939-8084, Japan Toyama Science Museum Toyama Japan; 3 Entomological Laboratory, Faculty of Agriculture, Ehime University, Tarumi 3-5-7, Matsuyama, 790-8566, Japan Ehime University Matsuyama Japan

**Keywords:** Aquatic beetles, Dytiscoidea, east Asia, Oriental Region, Palaearctic Region

## Abstract

The Japanese members of Haliplidae were reviewed and 13 species in two genera are recognized. A new species, *Haliplusmorii***sp. nov.** is described from Honshu; it is similar to *Haliplusjaponicus* Sharp, 1873, but belongs to a different subgenus. *Haliplusdiruptus* J. Balfour-Browne, 1946, **syn. nov.** is treated as a junior synonym of *Halipluskotoshonis* Kano & Kamiya, 1931. The records of *Haliplusdavidi* Vondel, 1991 from Japan are regarded as misidentifications of *H.kotoshonis*. *Haliplusbasinotatuslatiusculus* Nakane, 1985, **syn. nov.** is treated as a junior synonym of *H.basinotatus*. *Haliplusangustifrons* Régimbart, 1892 known from south and southeast Asia, is newly recorded from Japan.

## ﻿Introduction

The Japanese Haliplidae includes 11 species in two genera (Nakajima et al. 2020). Two species of the genus *Peltodytes* Régimbart, 1879 are recorded (Kamiya 1936) but the genus *Haliplus* Latreille, 1802 is one of the most diverse of the family. Among the Japanese *Haliplus* that have been reviewed by Nakane (1963a, 1963b, 1985) and Satô (1984, 1985), there are disagreements in the taxonomic assignments of some species. Since then, van Vondel (1991, 1995, 1998, 2003a, 2003b, 2019) and van Vondel et al. (2006) have extensively examined the Old World species, and the elucidation of Asian species has progressed. However, for Japanese species, unresolved problems remain due to confusion between species whose type specimens may have disappeared (Mori 2007). As a result of re-examination of Japanese Haliplidae, 13 species in two genera, including one new species and one newly recorded species, were found, and are described and illustrated. Additionally, a review was conducted on Japanese Haliplidae, and their distributions, larval stages, and ecological notes have been added.

## ﻿Materials and methods

Specimens studied herein are deposited at the following institutions and collections:

**EUMJ**Ehime University, Matsuyama, Japan [Hiroyuki Yoshitomi];

**HIPC** Hiroaki Iketake Private Collection, Nagoya, Japan;

**HOWP** Hoshizaki Institute for Wildlife Protection, Izumo, Japan [Masakazu Hayashi];

**IIM** Ishikawa Insect Museum, Hakusan, Japan [Kohei Watanabe];

**KAPC** Koji Arai Private Collection, Ranzan, Japan;

**KPMNH**Kanagawa Prefectural Museum of Natural History, Odawara, Japan [Kyohei Watanabe];

**KWPC** Kohei Watanabe Private Collection, Hakusan, Japan;

**RUMF** Ryukyu University Museum Fujukan, Nishihara, Japan [Takeshi Sasaki];

**TFPC** Takuya Fukuzawa Private Collection, Takao, Japan;

**TIPC** Tomofumi Iwata Private Collection, Toyama, Japan;

**TPM** Tochigi Prefectural Museum, Utsunomiya, Japan [Takashi Kurihara].

Minute structures on body surface of the new species were photographed under a scanning electron microscope (JCM-6000 Neoscope, JEOL Ltd., Tokyo, Japan). Male genitalia of the new species were photographed under a light microscope, Nikon Eclipse E600 with the digital camera (Digital Camera DS-L2, Nikon Ltd., Tokyo, Japan). The digital photographs were made by focus stacking, using a digital image processing software, Adobe Photoshop CS4 for Macintosh.

Stereoscopic (Leica S8 APO, Leica Microsystems Ltd., Wetzlar, Germany) and digital (HiROX KH-1300, HiROX Co. Ltd., Tokyo, Japan) microscopes were used to observe each part of the specimens except for the new species. Photographs were taken of the whole body, head, prosternal process, and male genitalia (penis, right and left paramere). Approximately ten images of the whole body were taken using a microscope camera (Digital Camera Unit DS-Fi1, Nikon Ltd., Tokyo, Japan) attached to a stereoscopic microscope, and focus stacking used to combine images using the image processing software CombineZM. The head and male genitalia were removed with tweezers after softening the dried specimens. The male genitalia were then placed in a 10% KOH solution for several hours to dissolve the flesh, and dissected under a stereomicroscope (Leica S8 APO).

The measurement abbreviations used in the text are shown below. The average is given in parenthesis after the range.

**BT** (body thickness): maximum thickness of body height;

**CED** (compound eyes distance): minimum distance between eyes;

**EL** (elytral length): maximum length of the elytron (measured on the line of suture);

**EW** (elytral width): maximum width of the elytra (two elytra);

**HW** (head width): maximum width of the head (including compound eyes);

**PL** (pronotal length): maximum length of pronotum;

**PW** (pronotal width): maximum width of pronotum;

**TL** total length (PL+EL).

Dried specimens were observed from the dorsal (HW, CED, PL, PW, EL, EW) and lateral (BT) views, and the maximum length at each measurement site was measured. For observation and measurement, a stereomicroscope (Olympus SZ40, Olympus Corporation, Tokyo, Japan) was used for PL, PW, EL, EW, and BT, and a stereomicroscope (Leica S8 APO) was used for HW and CED.

**Figure 1.**
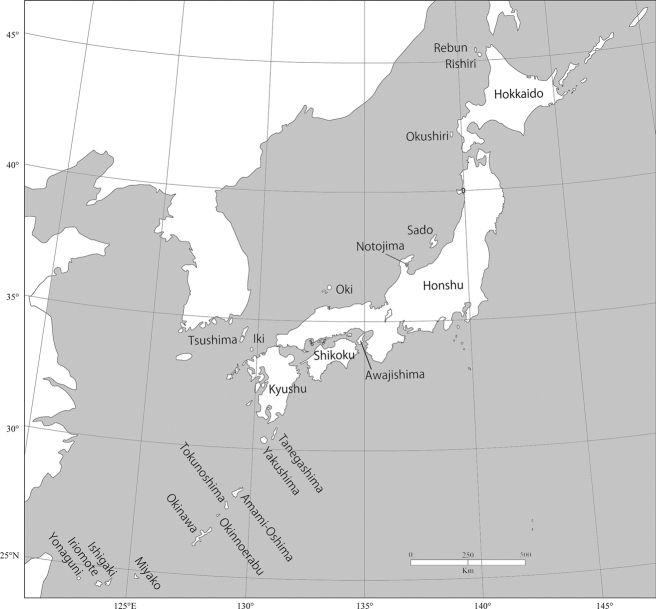
Map showing the island names included in this paper.

The terminology of body characters is followed by van Vondel (1991, 1992, 2019) and van Vondel et al. (2006). Fig. 1 shows the island names of Japan included in this paper.

## ﻿Taxonomic accounts

### ﻿Genus *Peltodytes* Régimbart, 1879

#### ﻿ Peltodytes intermedius

Taxon classificationAnimaliaColeopteraHaliplidae

(Sharp,1873)

2B82DB86-0F6F-541F-9B76-E0B4216F3D5D

**Figure 2.**
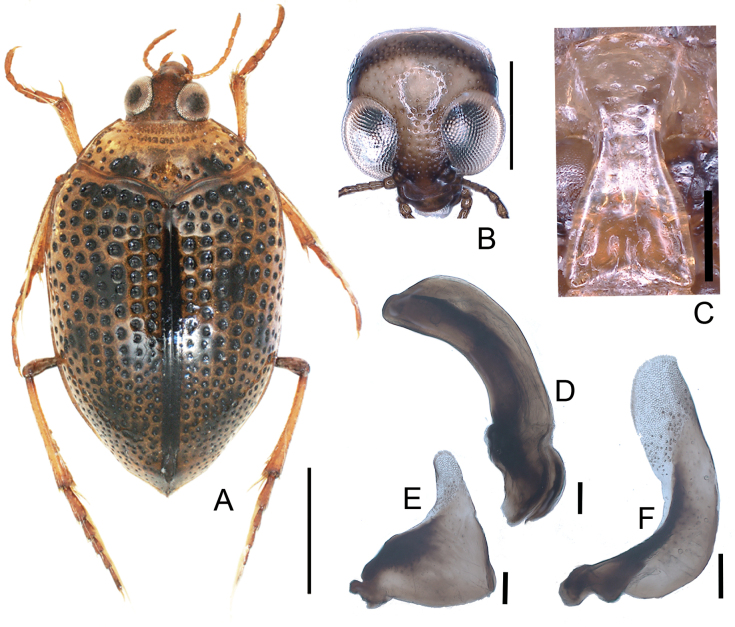
*Peltodytesintermedius***A** habitus **B** head **C** prosternal process **D** penis **E** left paramere **F** right paramere. Scale bars: 1.0 mm (**A**); 0.5 mm (**B**); 0.25 mm (**C**); 0.1 mm (**D–F**).

Figs 2 , 18A Japanese name: Kogashira-mizumushi

Cnemidotus intermedius Sharp, 1873: 55.
Peltodytes intermedius : Kamiya 1936: 39; 1951: 122; Satô 1985: 181; Nakane 1963a: 55; 1987: 27; Matsui 1992: 2; Vondel 1995: 124; 2003a: 33; Takahashi 1998: 6; Dejima 2007: 83; 2010: 43; Tomisawa 2012: 42; Yoshitomi 2014: 25; Matsuo and Fukagawa 2016: 50; Mitamura et al. 2017: 136; Hayashi and Kadowaki 2019: 25; Nakajima et al. 2020: 19; Nakamine and Nakamine 2021: 2.

##### Material examined.

1 ex., Ehime Prefecture: Hakata-jima, 14–15. VIII.1997, H. Nakanishi leg. (EUMJ); 1 ex., Ehime Prefecture: Iwagi-jima, 16.VIII.1997, H. Nakanishi leg. (EUMJ); 1 ex., Ehime Prefecture: Ômi-shima, 13.VIII.1997, H. Nakanishi leg. (EUMJ); 3 exs., Ehime Prefecture: Ôshima, 23.V.1998, H. Nakanishi leg. (EUMJ); 1 ex., Ehime Prefecture: Ôshima, 13.VIII.1997, H. Nakanishi leg. (EUMJ); 1 ex., Kagoshima Prefecture: Chaban, Yoron-to, 10.VIII.1958, S. Ueno & Y. Morimoto leg. (EUMJ). Other specimens are listed in Suppl. material 1.

##### Measurements

**(*n* = 10).**TL 3.38–4.82 (4.48) mm; HW 0.69–0.78 (0.73) mm; CED 0.19–0.23 (0.21) mm; PL 0.62–0.71 (0.67) mm; PW 1.45–1.64 (1.52) mm; EL 2.34–2.67 (2.45) mm; EW 1.86–2.16 (1.98) mm; BT 1.41–1.65 (1.53) mm; HW/CED 3.22–3.83 (3.49); PW/PL 2.18–2.35 (2.27); EL/EW 1.17–1.27 (1.22).

##### Biology.

This species typically inhabits stagnant water environments such as ponds, paddies, and swamps (Nakajima et al. 2020). The larvae primarily feed on Zygnemataceae algae. The adults were collected using a sweep net in shallow water or captured by a light trap. A pupal chamber was observed within the mud in rearing condition (Hayashi 2015).

##### Immature stages.

The larva was described by Fukuda et al. (1959) and illustrated in color by Mitamura et al. (2017) and Nakajima et al. (2020).

##### DNA barcodes.

The sequences of COI (Cox1) gene of Japanese specimens are deposited in DNA Data Bank of Japan (DDBJ) (Hayashi and Ooi 2022): Aomori Prefecture (LC633200), Shimane Prefecture (LC633201–LC633205).

##### Distribution.

Japan: Hokkaido, Honshu, Shikoku, Kyushu, Sado, Izu-shoto, Noto-jima of Nanao-wan in Ishikawa, Oki, Awaji-shima, Shodo-shima, Te-jima in Kagawa, Geiyo-shoto in Seto-naikai (new records) (Iwagi-jima, Ômishima, Hakata-jima, Ôshima), Tsushima, Iki, Hirado-jima in Nagasaki, Goto-retto, Taka-shima of Imari-wan in Nagasaki, Tobase-jima of Yatsushiro-kai in Kumamoto, Amakusa-shoto in Kumamoto, Nansei shoto (Tanegashima, Yoron-jima: new record); Korea, China, Taiwan, Far East of Russia.

#### ﻿ Peltodytes sinensis

Taxon classificationAnimaliaColeopteraHaliplidae

(Hope, 1845)

32AF777E-05B8-50F3-9EC8-B0D5EC5890F2

**Figure 3.**
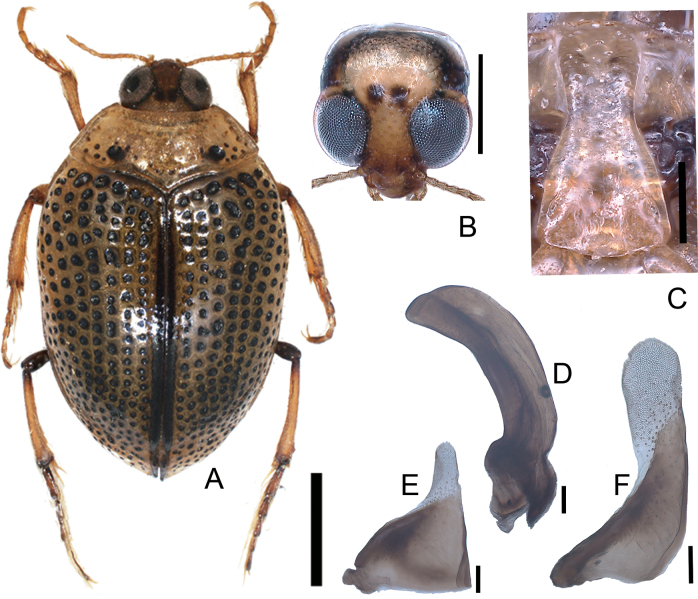
*Peltodytessinensis***A** habitus **B** head **C** prosternal process **D** penis **E** left paramere **F** right paramere. Scale bars: 1.0 mm (**A**); 0.5 mm (**B**); 0.25 mm (**C**); 0.1 mm (**D–F**).

Figs 3 , 18B Japanese name: Shina-kogashira-mizumushi

Haliplus sinensis Hope, 1845: 15.
Peltodytes sinensis : Kamiya 1936: 40; Satô 1985: 181; Nakane 1987: 27; Vondel 1995: 124; 2003a: 33; Aoyagi 2011a: 99; 2011b: 107; Matsuo and Fukagawa 2016: 51; Mitamura et al. 2017: 137; Aoyagi 2019: 34; Nakajima et al. 2020: 18; Aoyagi 2020: 87; Uchida et al. 2021: 183.

##### Material examined.

6 exs., Kagoshima Prefecture: Ohtsukan, Okinoerabu-jima, 3.VIII.1958, S. Ueno & Y. Morimoto leg. (EUMJ); 1 ex., Kagoshima Prefecture: Uchida, near, Taniyama, Okinoerabu-jima, 4.VIII.1958, S. Ueno & Y. Morimoto leg. (EUMJ). Other specimens are listed in Suppl. material 1.

##### Measurements

**(*n* = 10).**TL 3.06–5.21 (4.76) mm; HW 0.75–0.82 (0.78) mm; CED 0.20–0.25 (0.22) mm; PL 0.63–0.80 (0.71) mm; PW 1.46–1.68 (1.58) mm; EL 2.43–2.76 (2.58) mm; EW 1.91–2.30 (2.12) mm; BT 1.46–1.76 (1.62) mm; HW/CED 3.26–3.73 (3.49); PW/PL 2.01–2.40 (2.21); EL/EW 1.17–1.28 (1.21).

##### Biology.

This species usually lives in still, shallow waters such as ponds and paddies, and the adults were collected by sweep netting in shallow water and using light traps (Nakajima et al. 2020).

##### Immature stages.

Unknown.

##### Distribution.

Japan: Tsushima, Nansei shoto (Nakano-shima and Takara-jima of Tokara-retto, Amami-Ôshima, Kakeroma-jima, Tokuno-shima, Okinoerabu-jima (new record), Yoron-jima, Okinawa-jima, Yagaji-shima in Okinawa, Miyagi-jima in Okinawa, Iheya-jima, Kume-jima, Miyako-jima, Ikema-jima in Okinawa, Irabu-jima in Okinawa, Ishigaki-jima, Iriomote-jima, Yonaguni-jima); Korea, China, Taiwan, SE Asia.

### ﻿Genus *Haliplus* Latreille, 1802

#### Subgenus ﻿Nipponiplus Vondel, 2019

##### ﻿ Haliplus (Nipponiplus) japonicus

Taxon classificationAnimaliaColeopteraHaliplidae

Sharp, 1873

F39106A8-C760-56BD-9C54-9C619CF37315

**Figure 4.**
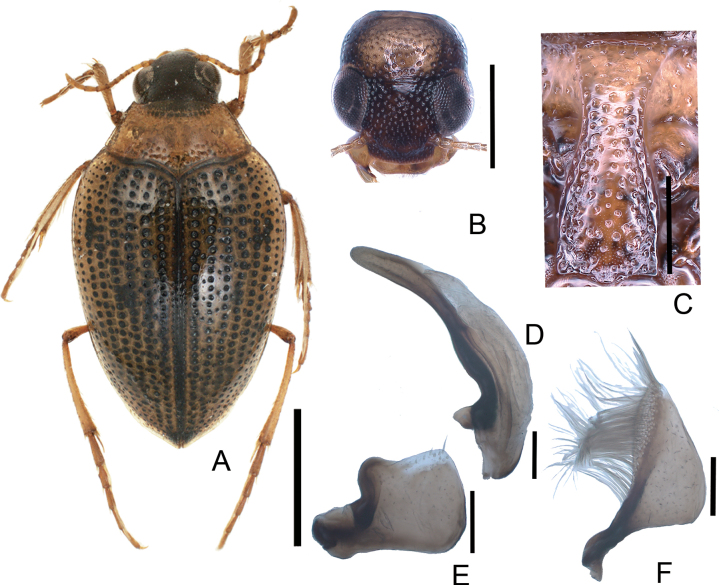
*Haliplusjaponicus***A** habitus **B** head **C** prosternal process **D** penis **E** left paramere **F** right paramere. Scale bars: 1.0 mm (**A**); 0.5 mm (**B**); 0.25 mm (**C**); 0.1 mm (**D–F**).

Figs 4 , 18C Japanese name: Kubiboso-kogashira-mizumushi

Haliplus japonicus Sharp, 1873: 55. Kamiya 1936: 44; Satô 1985: 181; Nakane 1963a: 55; 1985: 62; 1987: 29; Matsui 1992: 2; Vondel 1995: 122; Tomisawa 2012: 42; Matsuo and Fukagawa 2016: 51; Mitamura et al. 2017: 138; Hayashi and Kadowaki 2019: 25; Nakajima et al. 2020: 20.
Haliplus (Haliplus) japonicus : Satô, 1984: 1; Vondel 2003a: 31; Vondel et al. 2006: 250.
Haliplus (Nipponiplus) japonicus : Vondel 2019: 22.
Haliplus brevior Nakane, 1963a: 55. Nakane 1985: 62; 1987: 29. [synonymized with H.minutus by Satô (1984) but with H.japonicus by van Vondel et al. (2006)]

###### Material examined.

Specimens examined in this study are listed in Suppl. material 1.

###### Measurements

**(*n* = 10).**TL 2.69–4.08 (3.79) mm; HW 0.62–0.69 (0.67) mm; CED 0.29–0.32 (0.30) mm; PL 0.55–0.66 (0.62) mm; PW 1.05–1.20 (1.12) mm; EL 1.99–2.20 (2.10) mm; EW 1.50–1.71 (1.60) mm; BT 1.12–1.28 (1.19) mm; HW/CED 2.12–2.35 (2.24); PW/PL 1.65–2.04 (1.76); EL/EW 1.25–1.34 (1.32).

###### Biology.

This species usually lives in fresh waters with abundant aquatic plants such as ponds, paddies, and streams (Nakajima et al. 2020). The larvae eat Zygnemataceae algae (Hayashi 2015). The adults were collected by sweep netting in shallow waters and are rarely attracted by light traps (Hayashi 2015). The pupation in mud with a pupal chamber was accomplished by laboratory rearing (Hayashi 2015).

###### Immature stages.

The color photographs were provided by Mitamura et al. (2017) and Nakajima et al. (2020).

###### Distribution.

Japan: Hokkaido, Honshu, Shikoku, Kyushu, Noto-jima, Oki, Goto-retto, Taka-shima, Amakusa-shoto; China, Far East Russia.

##### ﻿ Haliplus (Nipponiplus) regimbarti

Taxon classificationAnimaliaColeopteraHaliplidae

Zaitzev, 1908

F39C7D3A-6F7A-55F8-8355-1172DCF7A0F6

**Figure 5.**
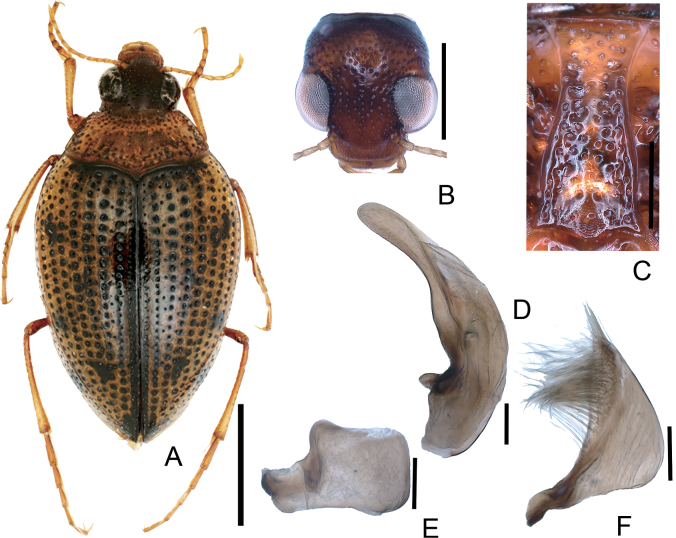
*Haliplusregimbarti***A** habitus **B** head **C** prosternal process **D** penis **E** left paramere **F** right paramere. Scale bars: 1.0 mm **A**; 0.5 mm (**B**); 0.25 mm (**C**); 0.1 mm (**D–F**).

Figs 5 , 18D Japanese name: Taiwan-kogashira-mizumushi

Haliplus brevis Wehncke, 1880: 7. [nec Stephens 1828]
Haliplus regimbarti Zaitzev, 1908: 122. [replacement name for Haliplusbrevis Wehncke, 1880]
Haliplus regimbarti : Vondel 1995: 122; Nakajima et al. 2020: 21.
Haliplus (Haliplus) regimbarti : Vondel et al. 2006: 257; Iwata 2016: 100.
Haliplus sauteri Zimmermann, 1924: 130. [synonymized by van Vondel 1995]

###### Material examined.

Specimens examined in this study are listed in Suppl. material 1.

###### Measurements

**(*n* = 4).**TL 2.87–4.44 (4.02) mm; HW 0.68–0.74 (0.72) mm; CED 0.31–0.34 (0.32) mm; PL 0.65–0.71 (0.68) mm; PW 1.23–1.35 (1.30) mm; EL 2.22–2.35 (2.31) mm; EW 1.76–1.90 (1.85) mm; BT 1.26–1.39 (1.35) mm; HW/CED 2.18–2.34 (2.23); PW/PL 1.84–1.98 (1.92); EL/EW 1.22–1.26 (1.25).

###### Biology.

This species was collected from a small pond with submerged water plants (Iwata 2016) and a narrow stream with flourishing water plants flowing beside paddy fields (Nakajima et al. 2020).

###### Immature stages.

Unknown.

###### Distribution.

Japan: Nansei shoto (Yonaguni-jima); Taiwan, south China.

#### Subgenus ﻿Haliplus Latreille, 1802

##### ﻿ Haliplus (Haliplus) kamiyai

Taxon classificationAnimaliaColeopteraHaliplidae

Nakane, 1963

C4714807-16CE-5E2A-877B-3AA3310A3005

**Figure 6.**
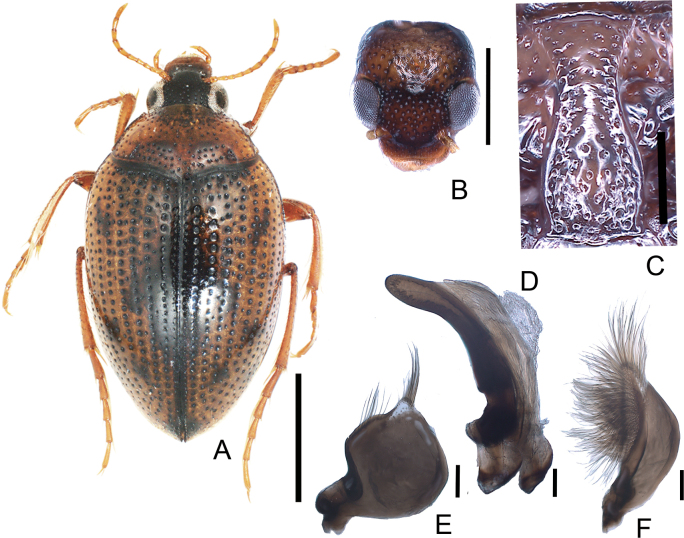
*Halipluskamiyai***A** habitus **B** head **C** prosternal process **D** penis **E** left paramere **F** right paramere. Scale bars: 1.0 mm (**A**); 0.5 mm (**B**); 0.25 mm (**C**); 0.1 mm (**D–F**).

Figs 6 , 18D Japanese name: Kamiya-kogashira-mizumushi

Haliplus kamiyai Nakane,1963b: 25. Nakane 1963a: 55; 1985: 62; 1987: 29; Mitamura et al. 2017: 141; Nakajima et al. 2020: 20.
Haliplus (Haliplus) kamiyai : Vondel et al. 2006: 252.

###### Material examined.

Specimens examined in this study are listed in Suppl. material 1.

###### Measurements

**(*n* = 10).**TL 2.64–4.24 (3.84) mm; HW 0.61–0.71 (0.66) mm; CED 0.34–0.36 (0.35) mm; PL 0.60–0.67 (0.64) mm; PW 1.06–1.25 (1.16) mm; EL 1.85–2.28 (2.08) mm; EW 1.46–1.71 (1.60) mm; BT 1.16–1.33 (1.24) mm; HW/CED 1.81–2.09 (1.89); PW/PL 1.76–1.90 (1.79); EL/EW 1.26–1.33 (1.29).

###### Biology.

This species lives in still water with abundant aquatic plants such as, ponds, paddies, and streams (Nakajima et al. 2020). The larvae feed on Zygnemataceae algae (Watanabe and Yamasaki 2020).

###### Immature stages.

Color photographs were provided by Watanabe and Yamasaki (2020).

###### Distribution.

Japan: Honshu (Tohoku and Kanto Regions).

##### ﻿ Haliplus (Haliplus) morii sp. nov.

Taxon classificationAnimaliaColeopteraHaliplidae

B5B9833B-4EA7-57C0-8BD9-0836F70B3FCE

https://zoobank.org/71E771A3-91A0-4F75-885E-4D1789CBCD8F

**Figure 7.**
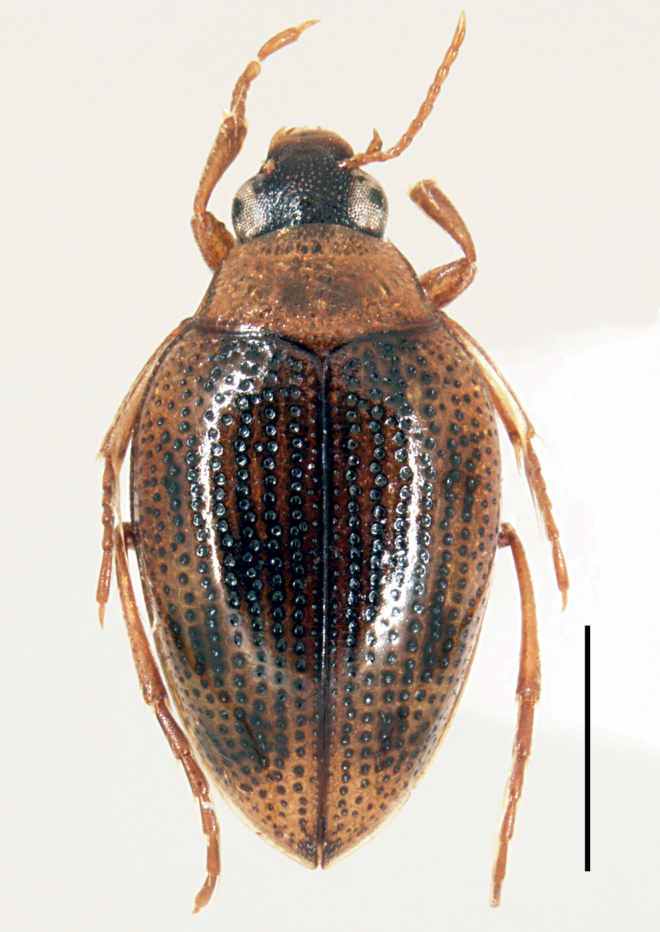
*Haliplusmorii* sp. nov. habitus. Scale bar: 1.0 mm.

**Figure 8.**
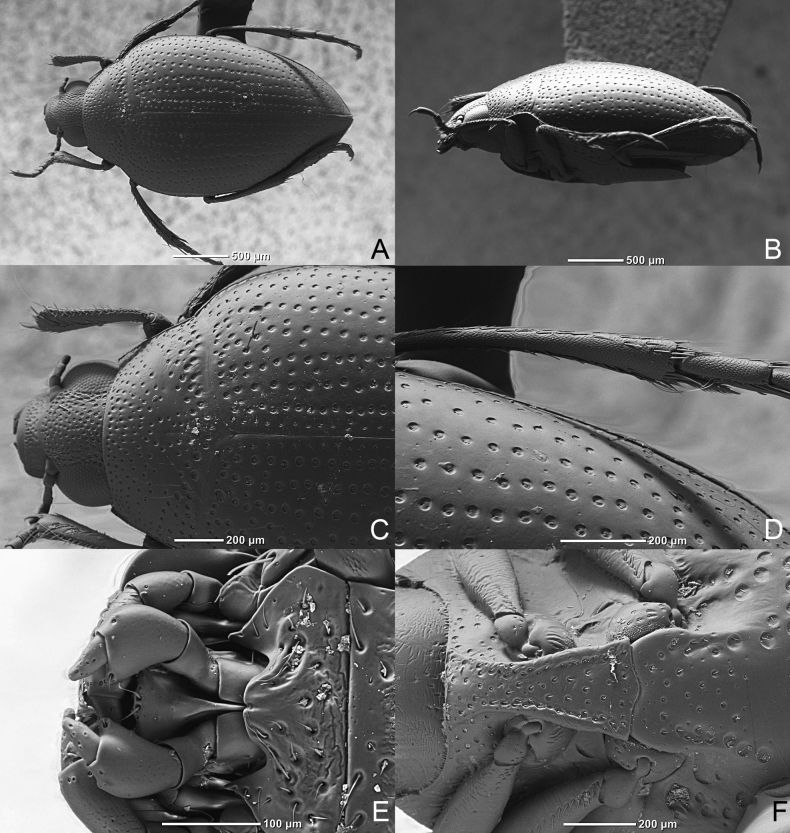
*Haliplusmorii* sp. nov. SEM **A** habitus **B** lateral side **C** apical half of body **D** right hind leg **E** labium **F** prosternal process.

**Figure 9.**
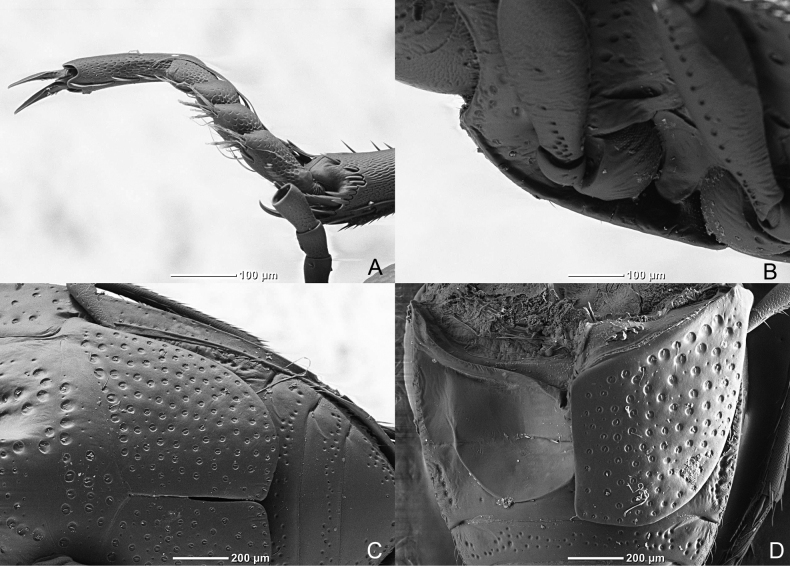
*Haliplusmorii* sp. nov. SEM **A** left pro leg **B** prosternal process **C** metacoxa **D** base of abdominal segments.

**Figure 10.**
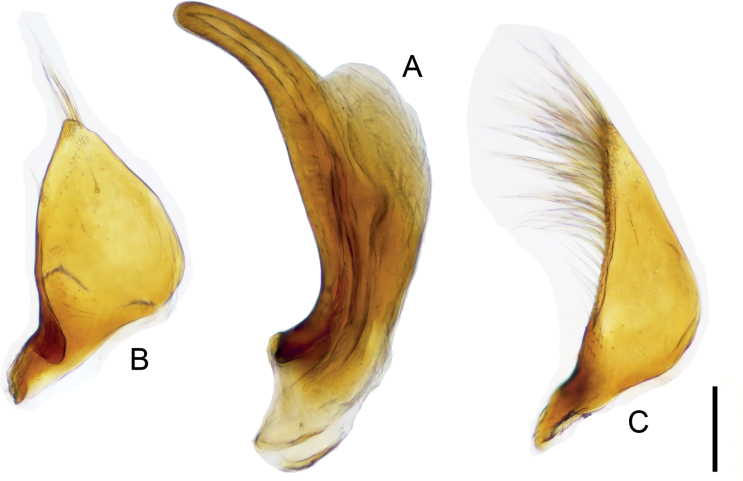
*Haliplusmorii* sp. nov. male genitalia **A** penis **B** left paramere **C** right paramere. Scale bar: 0.1 mm.

Figs 7 , 8 , 9 , 10 , 18D Japanese name: Mizonashi-kogashira-mizumushi

###### Type series.

***Holotype*** (Fig. 7) [EUMJ] 1 male Ochifushi, Yuza-machi, Yamagata Pref., Japan, 21.II.1993, M. Takahashi leg. ***Paratypes*** [EUMJ, HOWP] 11 exs. (male and female) Same data as holotype.

###### Description.

TL 3.0–3.3 mm, EW 1.5–1.6 mm. Body oval, tapering backwards, widest before the middle (Fig. 7).

***Head*.** Black, strongly and densely punctured, labrum dark yellow. Distance between eyes 1.7–2.0× width of one eye. Antennae yellow. Palpi yellow.

***Pronotum*.** Yellow to yellowish-brown. Plicae lacking (Fig. 8C). Coarsely punctate near base. Lateral sides margined, nearly straight.

***Elytra*.** Yellow to yellow-brown, dark interrupted lines on primary puncture rows, darkened along suture, sometime indistinct marks connecting primary puncture rows. Completely margined. Primary puncture rows moderately strong, dense in first rows, ~ 36 or 37 punctures in first row. Secondary punctures moderately strong and dense along suture, moderately strong and sparse on intervals. All punctures darkened.

***Ventral side*.** Yellow-brown, legs yellow-brown, slightly darkened towards coxae, elytral epipleura yellow-brown with strong punctures, reaching to sixth sternite. Prosternal process narrowed near coxae, grooved along each side, anterior edge weakly margined, moderately strongly punctured. Metasternal process flat or even slightly bulbous with a row of strong punctures on each side, else moderately punctured, emarginate in apical margin (Fig. 8F). Metacoxal plates reaching to fifth sternite, moderately strongly punctured, near suture weakly punctured, row of setae on posterior edge (Fig. 9C, D). Third and fourth sternites not fused with well indicated depression and around ridge. Fifth and sixth sternite with sparse transverse puncture row, last sternite weakly punctured in apical part. No setiferous striole on dorsal face of hind tibia, longer tibial spur of hind legs 2⁄3 length of first tarsal segment (Fig. 8D).

***Male genitalia*.** Penis carved in apical and basal part; slender and round in apex (Fig. 10A). Right paramere triangular, apex with long setae and lacking small segment (Fig. 10C).

###### Similar species.

This species may be confused with *H.japonicus*, but the latter has plicae on the pronotum.

###### Biology.

The type locality is dominated by rice paddy fields in the plains.

###### Distribution.

Japan (Tohoku Region).

###### Etymology.

The species name is dedicated to Mr. Masato Mori, who first noticed the existence of this species.

##### ﻿ Haliplus (Haliplus) simplex

Taxon classificationAnimaliaColeopteraHaliplidae

Clark, 1863

F9665DA6-1D3B-5E55-9DEF-55C963DF9D1C

**Figure 11.**
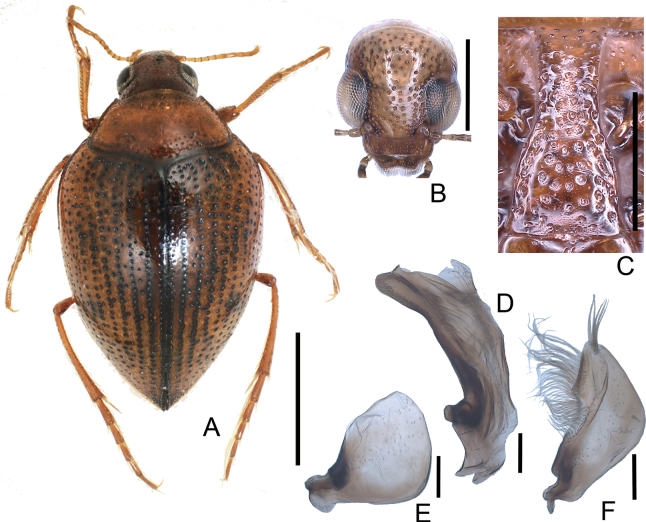
*Haliplussimplex***A** habitus **B** head **C** prosternal process **D** penis **E** left paramere **F** right paramere. Scale bars: 1.0 mm (**A**); 0.5 mm (**B**); 0.25 mm (**C**); 0.1 mm (**D–F**).

Figs 11 , 18E Japanese name: Chibi-kogashira-mizumushi

Haliplus simplex Clark, 1863: 419. Vondel 1995: 123; Matsuo and Fukagawa 2016: 51; Mitamura et al. 2017: 142; Nakajima et al. 2020: 21.
Haliplus minutus Takizawa, 1931: 140. Kamiya 1936: 45; Satô 1985: 181; Nakane 1985: 62. [synonymized by van Vondel et al. (2006)]
Haliplus (Haliplus) minutus : Satô 1984: 1; Vondel 2003a: 31; Vondel et al. 2006: 265.

###### Material examined.

Specimens examined in this study are listed in Suppl. material 1.

###### Measurements

**(*n* = 10).**TL 2.49–3.75 (3.54) mm; HW 0.62–0.66 (0.64) mm; CED 0.32–0.35 (0.33) mm; PL 0.57–0.62 (0.59) mm; PW 1.04–1.14 (1.10) mm; EL 1.86–1.99 (1.92) mm; EW 1.50–1.61 (1.55) mm; BT 1.14–1.19 (1.15) mm; HW/CED 1.86–2.02 (1.92); PW/PL 1.75–1.94 (1.84); EL/EW 1.21–1.28 (1.24).

###### Biology.

This species typically inhabits stagnant water environments such as ponds, paddies, and swamps, and the adults were collected by sweep netting in shallow water (Nakajima et al. 2020).

###### Immature stages.

Unknown.

###### Distribution.

Japan: Hokkaido, Honshu (Tohoku Region), Tsushima; Korea, China, Far East of Russia.

#### ﻿Subgenus Liaphlus Guignot, 1928

##### ﻿ Haliplus (Liaphlus) angustifrons

Taxon classificationAnimaliaColeopteraHaliplidae

Régimbart, 1892

F7C85831-CA8F-57D6-B7A7-CFC34F9229A8

**Figure 12.**
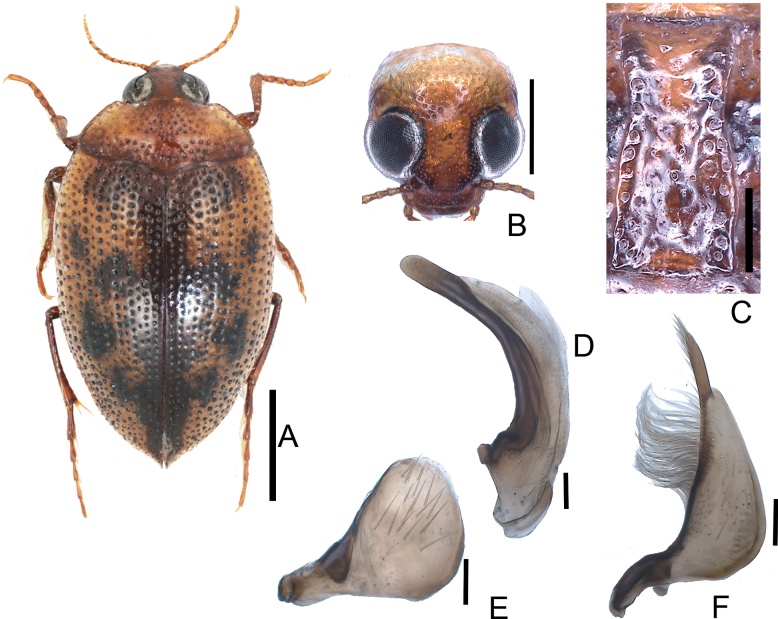
*Haliplusangustifrons***A** habitus **B** head **C** prosternal process **D** penis **E** left paramere **F** right paramere. Scale bars: 1.0 mm (**A**); 0.5 mm (**B**); 0.25 mm (**C**); 0.1 mm (**D–F**).

Figs 12 , 18E Japanese name: Usucha-kogashira-mizumushi

Haliplus angustifrons Régimbart, 1892: 112. van Vondel 1993: 292; Sheth et al. 2016: 361.
Haliplus kotoshonis : Vondel, 1991: 113 [misidentification]; Aoyagi 2014: 192 [misidentification].

###### Material examined.

2 exs., Kagoshima Prefecture: Amagi, Amagi-cho, Ôshima-gun, Tokuno-shima, 1.XI.2010, H. Iketake leg. (HIPC); 27 exs., Okinawa Prefecture: Ohgimi, Ohgimi-son [Okinawa-jima], 19.III.2014, R. Okano leg. (EUMJ); 1 ex., Okinawa Prefecture: Nakama, Onna-son [Okinawa-jima], 30.X.2011, H. Iketake leg. (HIPC); 1 ex., Okinawa Prefecture: Onna-dam, Sokei, Ginoza-son [Okinawa-jima], 30.X.2011, H. Iketake leg. (HIPC); 6 exs., Okinawa Prefecture: Nuuha, Ohgimi-son [Okinawa-jima], 8.I.1989, Y. Abe & T. Abe leg. (KPMNH); 1 ex., Okinawa Prefecture: Gima, Kumejima-cho, 19.V.2007, Y. Kamite leg. (EUMJ); 1 ex., Okinawa Prefecture: Kanegusuku, Kumejima-cho, 20.V.2007, Y. Kamite leg. (EUMJ); 6 exs., Okinawa Prefecture: Uehara, Taketomi-cho (24.40°N, 123.79°E) [Iriomote-jima], 14.III.2017, T. Iwata leg. (TIPC); 1 ex., Okinawa Prefecture: Ishigaki-jima, 5.VI.1970, T. Hozumi leg. (EUMJ).

###### Measurements

**(*n* = 10).**TL 3.66–5.36 (4.98) mm; HW 0.80–0.87 (0.84) mm; CED 0.27–0.30 (0.28) mm; PL 0.76–0.82 (0.79) mm; PW 1.64–1.76 (1.68) mm; EL 2.67–2.86 (2.76) mm; EW 2.04–2.25 (2.14) mm; BT 1.48–1.69 (1.59) mm; HW/CED 2.85–3.07 (2.95); PW/PL 2.07–2.17 (2.12); EL/EW 1.27–1.32 (1.29).

###### Biology.

The above specimens were collected from small ponds.

###### Immature stages.

Unknown.

###### Discussion.

*Haliplusangustifrons* is widely distributed from south Asia to southeast Asia (van Vondel 1993; Sheth et al. 2016), but there are no records in east Asia. The pattern of dorsal marks, the shape of the prosternal process, and male genitalia are in agreement with the redescription of *H.angustifrons* (van Vondel 1993). The figures of “*H.kotoshonis*” given by van Vondel (1991, 1993) are not of true *H.kotoshonis*, and at least the records of Japanese specimens correspond to *H.angustifrons*.

###### Distribution.

Japan (new records): Nansei shoto (Tokuno-shima, Okinawa-jima, Iheya-jima, Kume-jima, Ishigaki-jima, Iriomote-jima); Pakistan, India, Sri Lanka, Nepal, Myanmar, Laos, Vietnam.

##### ﻿ Haliplus (Liaphlus) basinotatus

Taxon classificationAnimaliaColeopteraHaliplidae

Zimmermann, 1924

8F9A8BC9-B23E-50F4-8E3E-ADE9E523FFFE

**Figure 13.**
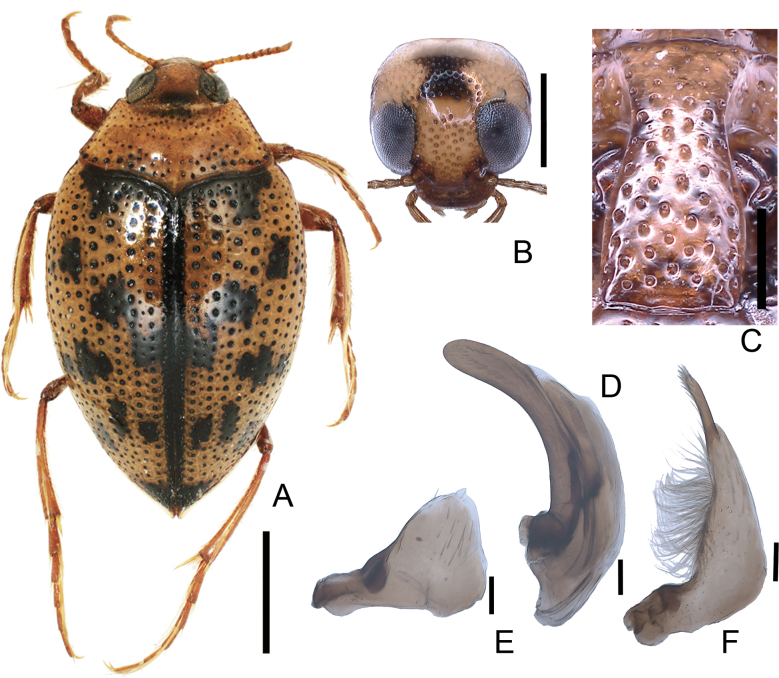
*Haliplusbasinotatus***A** habitus **B** head **C** prosternal process **D** penis **E** left paramere **F** right paramere. Scale bars: 1.0 mm (**A**); 0.5 mm (**B**); 0.25 mm (**C**); 0.1 mm (**D–F**).

Figs 13 , 18F Japanese name: Kurohoshi-kogashira-mizumushi

Haliplus basinotatus Zimmermann, 1924: 137. Nakane 1963a: 55; 1987: 30; Vondel 1995: 119; Matsuo and Fukagawa 2016: 52; Mitamura et al. 2017: 139; Hayashi and Kadowaki 2019: 25; Nakajima et al. 2020: 22; Watanabe 2021: 16.
Haliplus (Liaphlus) basinotatus : Vondel 2003a: 31.
Haliplus basinotatus latiusculus Nakane, 1985: 63. Syn. nov.

###### Material examined.

Specimens examined in this study are listed in Suppl. material 1.

###### Measurements

**(*n* = 10).**TL 3.30–5.34 (4.88) mm; HW 0.78–0.88 (0.83) mm; CED 0.30–0.38 (0.35) mm; PL 0.71–0.75 (0.74) mm; PW 1.43–1.59 (1.52) mm; EL 2.54–2.86 (2.66) mm; EW 1.95–2.17 (2.06) mm; BT 1.60–1.74 (1.65) mm; HW/CED 2.27–2.62 (2.35); PW/PL 1.95–2.14 (2.05); EL/EW 1.24–1.32 (1.27).

###### Biology.

The larvae eat Characeae algae, and the adults were collected by sweep on shallow water (Nakajima et al. 2020).

###### Immature stages.

A color illustration is given by Mitamura et al. (2017).

###### DNA barcodes.

The COI (Cox1) gene sequence of a Japanese specimen are deposited in DDBJ (Hayashi and Ooi 2022): Shimane Prefecture (LC633206).

###### Discussion.

Nakane (1985) described a subspecies for Japanese population, but we could not find any differences between the Asian (van Vondel 1995: fig. 53) and Japanese populations. In this paper we treat this subspecies as a junior synonym of nominotypical subspecies.

###### Distribution.

Japan: Hokkaido, Honshu, Shikoku, Kyushu, Oki, Tsushima; Korea, China, Far East Russia.

##### ﻿ Haliplus (Liaphlus) kotoshonis

Taxon classificationAnimaliaColeopteraHaliplidae

Kano & Kamiya, 1931

F1B9F79A-3CDF-538C-97C7-8C311848E4BC

**Figure 14.**
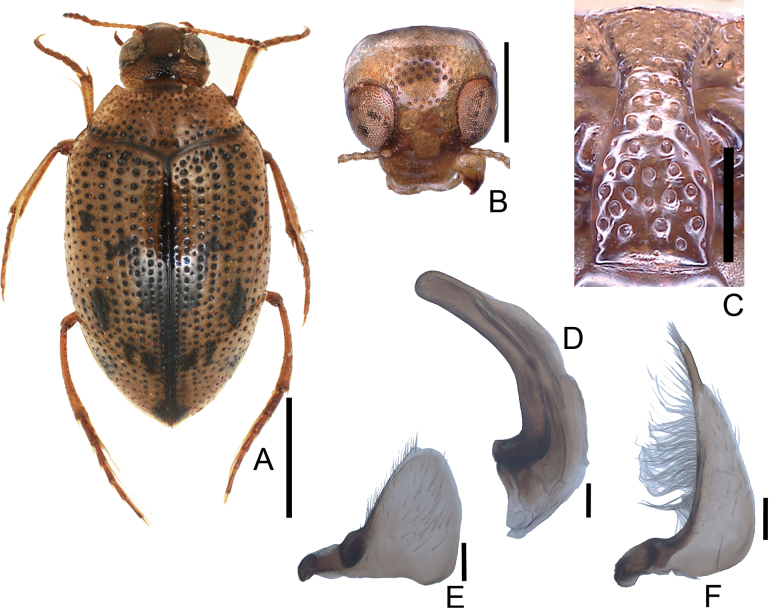
*Halipluskotoshonis***A** habitus **B** head **C** prosternal process **D** penis **E** left paramere **F** right paramere. Scale bars: 1.0 mm (**A**); 0.5 mm (**B**); 0.25 mm (**C**); 0.1 mm (**D–F**).

Figs 14 , 18F Japanese name: Koto-kogashira-mizumushi

Haliplus kotoshonis Kano & Kamiya, 1931: 2. Satô 1985: 181, pls 33–37; Nakane 1985: 63; 1987: 30; Nakajima et al. 2020: 21.
Haliplus (Liaphlus) kotoshonis : Satô 1984: 2.
Haliplus diruptus J. Balfour-Browne, 1946: 436. Vondel 1995: 120, figs 71–79. Syn. nov.
Haliplus davidi Vondel, 1991: 92. [synonymized with Haliplusdiruptus by van Vondel (2017)]

###### Material examined.

1 ex., Kagoshima Prefecture: Takara-jima, Tokara, 2.VII.1960, M. Satô leg. (EUMJ); 2 exs., Kagoshima Prefecture: Takara-jima, Tokara, 20.VII.1961, Y. Hama leg. (EUMJ); 7 exs., Kagoshima Prefecture: Takara-jima, Tokara, 2.VI.1962, M. Satô leg. (EUMJ); 2 exs., Okinawa Prefecture: Maesato, Ishigaki-jima, 4.IX.1975, T. Takahashi leg. (EUMJ); 1 ex., Okinawa Prefecture: Mt. Urabu-dake, Yonaguni-jima, 1.IV.1990, Y. Uchida leg. (EUMJ); 1 ex., Okinawa Prefecture: Shirahama, Iriomote-jima, 30.VI.2001, T. Nakamura leg. (TPM); 3 exs., Okinawa Prefecture: Okinawa-jima, Ryukyu, 26.VI.1995, T. Takara leg. (RUMF).

###### Measurements

**(*n* = 10).**TL 3.39–4.94 (4.47) mm; HW 0.74–0.87 (0.80) mm; CED 0.31–0.38 (0.33) mm; PL 0.65–0.79 (0.73) mm; PW 1.29–1.53 (1.42) mm; EL 2.30–2.62 (2.42) mm; EW 1.76–2.01 (1.88) mm; BT 1.39–1.70 (1.50) mm; HW/CED 2.30–2.56 (2.38); PW/PL 1.87–2.04 (1.95); EL/EW 1.23–1.32 (1.30).

###### Biology.

The above specimens were collected from small ponds.

###### Immature stages.

Unknown.

###### Discussion.

This species was described based on one specimen from “Kotosho” (Orchid Island), Taiwan (Kano and Kamiya 1931). The type specimen is likely to have been lost (Mita et al. 2015), but the black marks on the dorsum are unique and the original description and figures are recognizable. Nakane (1985, 1987, 1990) has repeatedly pointed out that *Haliplusdiruptus* J. Balfour-Browne, 1946 should be a junior synonym of the species, but a formal treatment has not been done. Judging from our investigation of the specimens and the description of van Vondel (1995), *H.diruptus* is treated as a junior synonym of this species in this paper. In addition, *Haliplusdavidi* Vondel, 1991 was recorded from Japan (van Vondel 1998, 2003a), but was later treated as a junior synonym of *H.diruptus* (van Vondel 2017); therefore, the record of *H.davidi* from Japan is not *H.diruptus* but *H.kotoshonis*.

This study revealed that specimens of *Haliplusangustifrons* Régimbart, 1892 were mixed in with the specimens identified as “*H.kotoshonis*” from Japan. In a typical individual, the dorsal marks can easily distinguish between the two species. In particular, this species has a black mark on head, but the *H.angustifrons* does not have such a mark.

###### Distribution.

Japan: Nansei shoto (Takara-jima of Tokara-retto, Okinawa-jima, Ishigaki-jima, Iriomote-jima, Yonaguni-jima); Taiwan, China, Vietnam, Myanmar.

##### ﻿ Haliplus (Liaphlus) eximius

Taxon classificationAnimaliaColeopteraHaliplidae

Clark, 1863

D25DD4FC-000A-5616-992D-6AED34C35F78

**Figure 15.**
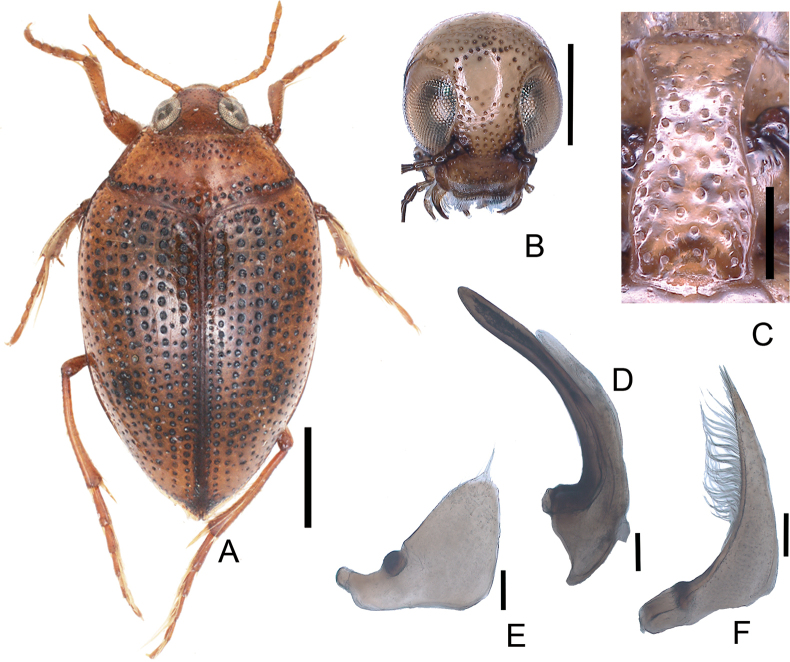
*Halipluseximius***A** habitus **B** head **C** prosternal process **D** penis **E** left paramere **F** right paramere. Scale bars: 1.0 mm (**A**); 0.5 mm (**B**); 0.25 mm (**C**); 0.1 mm (**D–F**).

Figs 15 , 19A Japanese name: Kiiro-kogashira-mizumushi

Haliplus eximius Clark,1863: 418. Kamiya 1936: 42; Satô 1985: 181; Nakane 1985: 63; 1987: 29; Matsui 1992: 2; Vondel 1995: 121; Dejima 2007: 84; Matsuo and Fukagawa 2016: 52; Mitamura et al. 2017: 141; Hayashi and Kadowaki 2019: 25; Nakajima et al. 2020: 23; Watanabe and Ohba 2022: 34.
Haliplus (Liaphlus) eximius : Satô 1984: 2; Vondel 1991: 97; 1993: 299; 2003a: 31.
Haliplus hiogoensis Kano & Kamiya, 1931: 1. Kamiya 1936: 44. [synonymized by Satô 1984]

###### Material examined.

1 ex., Ehime Prefecture: Ôshima, 13.VIII.1997, H. Nakanishi leg. (EUMJ). Other specimens examined in this study are listed in Suppl. material 1.

###### Measurements

**(*n* = 10).**TL 3.26–4.71 (4.38) mm; HW 0.70–0.78 (0.75) mm; CED 0.27–0.33 (0.30) mm; PL 0.69–0.75 (0.73) mm; PW 1.33–1.54 (1.46) mm; EL 2.10–2.53 (2.36) mm; EW 1.66–1.94 (1.85) mm; BT 1.31–1.51 (1.44) mm; HW/CED 2.23–2.70 (2.49); PW/PL 1.89–2.08 (2.03); EL/EW 1.24–1.32 (1.27).

###### Biology.

This species typically inhabits ponds, and the adults were collected by sweep nets over shallow water (Nakajima et al. 2020). The larvae eat Characeae algae (Yamazaki et al. 2020).

###### Immature stages.

The color photograph was shown by Kitano and Sano (2011).

###### DNA barcodes.

The COI (Cox1) gene sequence of one Japanese specimen were deposited in DDBJ (Hayashi and Ooi 2022): Shimane Prefecture (LC633207).

###### Distribution.

Japan: central to west Honshu, Shikoku, Kyushu, Oki, Hiro-shima in Kagawa, Geiyo-shoto (Ôshima: new record), Tsushima, Iki, Hirado-jima, Goto-retto, Amakusa-shoto, Nansei shoto (Yonaguni-jima); Korea, China, SE Asia.

##### ﻿ Haliplus (Liaphlus) ovalis

Taxon classificationAnimaliaColeopteraHaliplidae

Sharp, 1884

888272F4-E13D-592A-83DD-F4811F038D5A

**Figure 16.**
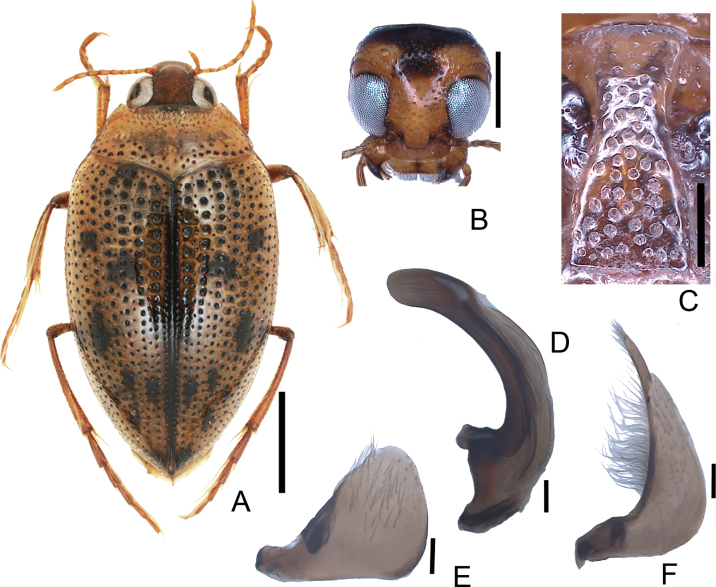
*Haliplusovalis***A** habitus **B** head **C** prosternal process **D** penis **E** left paramere**F** right paramere. Scale bars: 1.0 mm (**A**); 0.5 mm (**B**); 0.25 mm (**C**); 0.1 mm (**D–F**).

Figs 16 , 19B Japanese name: Hime-kogashira-mizumushi

Haliplus ovalis Sharp, 1884: 440. Kamiya 1936: 46; Satô 1985: 181; Nakane 1963a: 55; 1985: 63; 1987: 30; Matsuo and Fukagawa 2016: 52; Hayashi and Kadowaki 2019: 25; Nakajima et al. 2020: 23.
Haliplus (Liaphlus) ovalis : Satô 1984: 3; Vondel 1991: 125; 2003a: 32.

###### Material examined.

3 exs., Kagoshima Prefecture: Amagi, Amagi-cho, Ôshima-gun, Tokuno-shima, 1.XI.2010, H. Iketake leg. (HIPC); 1 ex., Niigata Prefecture: Ohura, Aikawa-machi, Sadoga-shima, 27–30. V. 1989, Y. Abe & T. Abe leg. (KPMNH); 11 exs., Kagoshima Prefecture: Ontsukan, Okinoerabu-jima, 3.VIII.1958, S. Ueno & Y. Morimoto leg. (EUMJ); 3 exs., Kagoshima Prefecture: Minzuki-ike, Okinoerabu-jima, 16.VIII.1958, S. Ueno & Y. Morimoto leg. (EUMJ); 12 exs., Kagoshima Prefecture: Okinoerabu-jima, 4.VIII.1958, S. Ueno & Y. Morimoto leg. (EUMJ).

###### Measurements

**(*n* = 10).**TL 3.80–5.97 (5.47) mm; HW 0.90–0.99 (0.95) mm; CED 0.37–0.43 (0.40) mm; PL 0.74–0.85 (0.80) mm; PW 1.57–1.75 (1.66) mm; EL 2.81–3.18 (3.06) mm; EW 2.10–2.38 (2.22) mm; BT 1.67–1.95 (1.79) mm; HW/CED 2.29–2.50 (2.44); PW/PL 1.99–2.16 (2.13); EL/EW 1.34–1.42 (1.39).

###### Biology.

This species typically inhabits ponds, and the adults were collected by sweep nets over shallow water and a light trap (Nakajima et al. 2020).

###### Immature stages.

The larva was illustrated by Satô and Yoshitomi (2018).

###### Discussion.

*Halipluschinensis* Falkenström, 1932 distributed in mainland China is closely similar to this species. van Vondel (1991) states that the distinguishing points of both species, *H.ovalis* and *H.chinensis*, are the distance between the eyes and the morphology of the apical curve of penis (smooth in *H.chinensis* and flexed in *H.ovalis*). But some individuals from Ryukyu (Okinoerabu-jima and Tokunoshima), Shikoku (Ehime Prefecture), and Honshu (Niigata Prefecture) have a smooth apex of the penis. Further detailed comparisons of both species are necessary.

###### DNA barcodes.

The sequences of COI (Cox1) gene of Japanese specimens are deposited in DDBJ (Hayashi and Ooi 2022): Shimane Prefecture (LC633208–LC633212).

###### Distribution.

Japan: Hokkaido, Honshu, Shikoku, Kyushu, Sado (new record), Oki, Goto-retto, Nansei shoto (Tokuno-shima, Okinoerabu-jima: new record).

##### ﻿ Haliplus (Liaphlus) sharpi

Taxon classificationAnimaliaColeopteraHaliplidae

Wehncke, 1880

9BFC5C7E-D6F1-5B1B-9F04-5BAD909899B9

**Figure 17.**
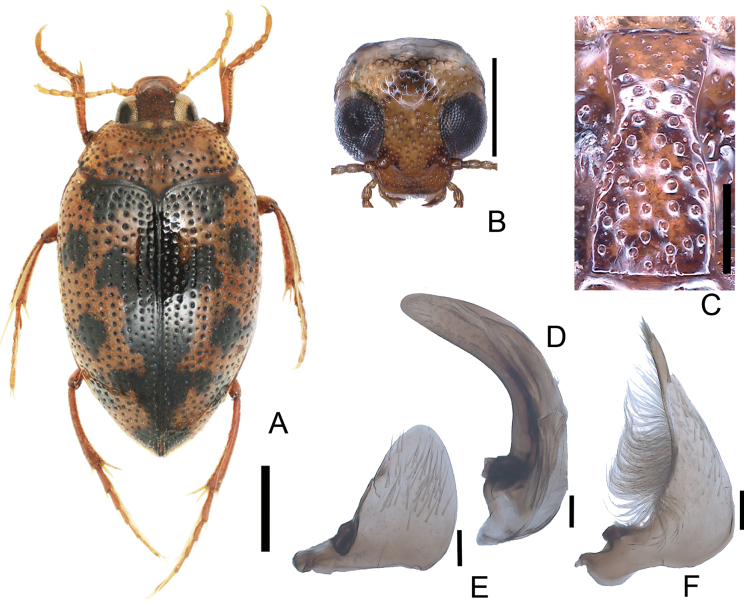
*Haliplussharpi***A** habitus **B** head **C** prosternal process **D** penis **E** left paramere **F** right paramere. Scale bars: 1.0 mm (**A**); 0.5 mm (**B**); 0.25 mm (**C**); 0.1 mm (**D–F**).

Figs 17 , 19C Japanese name: Madara-kogashira-mizumushi

Haliplus sharpi Wehncke, 1880: 74. Satô 1985: 181; Nakane 1985: 63; 1987: 30; Vondel 1995: 123; Matsuo and Fukagawa 2016: 53; Mitamura et al. 2017: 140; Hayashi and Kadowaki 2019: 25; Nakajima et al. 2020: 24; Nakamine and Nakamine 2021: 2; Imasaka et al. 2021: 71; Watanabe and Ohba 2022: 34.
Haliplus (Liaphlus) sharpi : Satô 1984: 3; Vondel 1991: 129; 1993: 313; 2003a: 32.
Haliplus simplex : Kamiya 1936: 48. [misidentification]
Haliplus tsukushiensis Yoshimura, 1932: 102. Nakane 1963a: 55. [synonymized by Satô 1984]
Haliplus holmeni Vondel, 1991: 109. [synonymized by van Vondel 2017]

###### Material examined.

10 exs., Niigata Prefecture: Ohura, Aikawa-machi, Sadoga-shima, 27–30. V.1989, Y. Abe & T. Abe leg. (KPMNH).

###### Measurements

**(*n* = 10).**TL 3.22–4.81 (4.48) mm; HW 0.71–0.81 (0.75) mm; CED 0.28–0.35 (0.31) mm; PL 0.70–0.80 (0.75) mm; PW 1.35–1.51 (1.44) mm; EL 2.30–2.52 (2.39) mm; EW 1.75–1.95 (1.87) mm; BT 1.40–1.56 (1.49) mm; HW/CED 2.25–2.69 (2.41); PW/PL 1.86–2.04 (1.95); EL/EW 1.23–1.33 (1.27).

###### Biology.

This species typically inhabits stagnant water environments such as ponds, paddies, and swamp (Nakanishi 2012; Watanabe and Hidaka 2013). The larvae feed on Characeae algae (Nakanishi 2012). The pupation occurred in the pupal chamber in laboratory rearing experiments (Nakanishi 2012).

###### Immature stages.

The color photographs were provided by Nakanishi (2012) and Mitamura et al. (2017).

**Figure 18.**
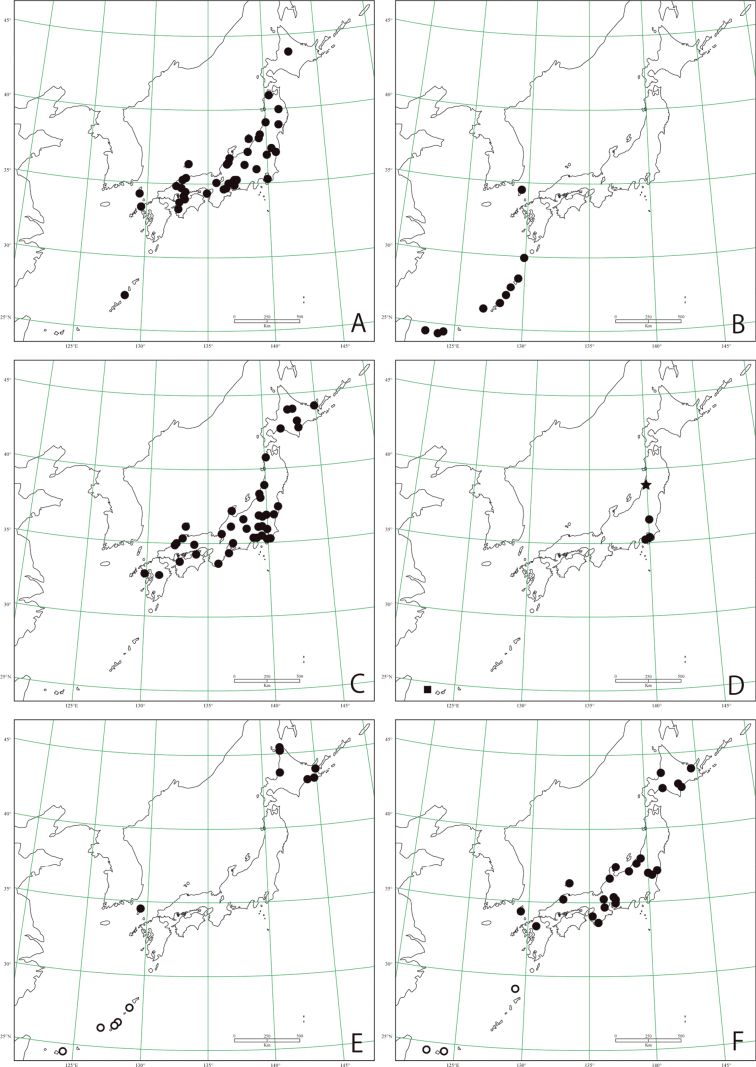
Distribution maps based on specimens examined **A***Peltodytesintermedius***B***Peltodytessinensis***C***Haliplusjaponicus***D***Halipluskamiyai* (circle), *Haliplusmorii* (star), *Haliplusregimbarti* (square) **E***Haliplussimplex* (black circle), *Haliplusangustifrons* (white circle) **F***Haliplusbasinotatus* (black circle), *Halipluskotoshonis* (white circle).

###### Distribution.

Japan: Hokkaido, Honshu, Shikoku, Kyushu, Sado (new record), Oki, Tsushima, Iki, Azuchi-ôshima in Nagasaki, Hirado-jima, Goto-retto, Koshikishima-retto, Nansei shoto (Tanegashima); Korea, China, Taiwan.

**Figure 19.**
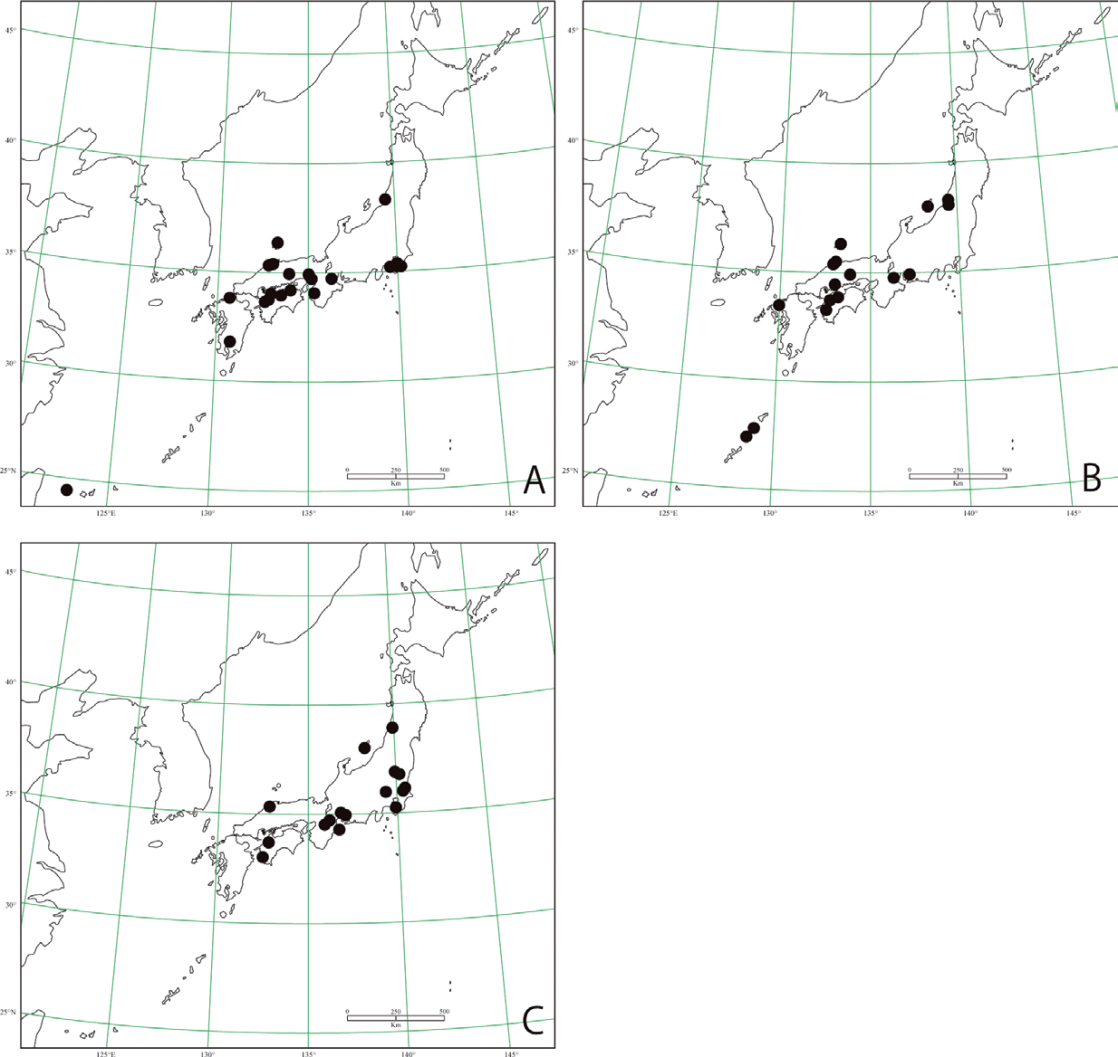
Distribution maps based on specimens examined **A***Halipluseximius***B***Haliplusovalis***C***Haliplussharpi*.

### ﻿Key to the species of *Haliplus* from Japan

Revised from Nakajima et al. 2020.

**Table:** 

1	Pronotum with plicae (a pair of short grooves on basal-middle part)	**2**
–	Pronotum without plicae (Fig. 8C)	**5**
2	Prosternal process without margin; front margin shorter than basal margin (Fig. 11C). Outline of elytra triangular (Fig. 11A). Penis robust in lateral view, apex with spines (Fig. 11D)	***H.simplex***
–	Prosternal process with margin; front margin as long as basal margin. Outline of elytra oval. Penis without apical spines in lateral view	**3**
3	Lateral sides of prosternal process slightly narrowing on base (Fig. 6C). Elytral marks developed, cruciform pattern on center (Fig. 6A). Penis curved nearly to apex and extremely narrowed (Fig. 6D)	***H.kamiyai***
–	Lateral sides of prosternal process slightly widened on base. Elytral marks not developed, small black spots on disc. Penis gently curved from middle to apex	**4**
4	Lateral surfaces of prosternal process with indistinct and narrow margins (Fig. 4C). Elytra gray (Fig. 6A). Penis slender at apex (Fig. 4D). Right paramere rounded at apex (Fig. 4F)	***H.japonicus***
–	Lateral surfaces of prosternal process with distinct and wide margins (Fig. 5C). Elytra yellowish colored (Fig. 5A). Penis robust at apex (Fig. 5E). Right paramere acute at apex (Fig. 5F)	***H.regimbarti***
5	Head, pronotum, and elytra without black mark (Fig. 15A, B). Penis entirely slender, apex acute (Fig. 15D)	***H.eximius***
–	Head, pronotum, and elytra with some black marks or entirely black. Penis entirely robust, apex rounded	**6**
6	Head entirely black (Fig. 7). Black marks on elytra indistinct (Fig. 7)	***H.morii* sp. nov.**
–	Head partly black or without black mark. Black marks on elytra distinct	**7**
7	Sutural black band usually reaches main 1^st^ interval on elytral base	**8**
–	Sutural black band barely reached main 1^st^ interval on elytral base	**9**
8	Head with black mark (Fig. 17B). Size of eye moderate (Fig. 17B). Black band on elytral base clear in outline (Fig. 17A). Central surface of prosternal process flat in apical half (Fig. 17C)	***H.sharpi***
–	Head without black mark (Fig. 12B). Size of eye large (Fig. 12B). Black band on elytral base unclear in outline (Fig. 12A). Central surface of prosternal process slightly depressed on apical half (Fig. 12C)	***H.angustifrons***
9	Black mark on head widened at base (Fig. 16B). Black marks on elytra arranged in diamond shape centrally; two black marks on 4^th^ interval, and one black mark on tip of 6^th^ interval (Fig. 16A)	***H.ovalis***
–	Black mark on head not widened at base. Black marks on elytra are aligned in straight line centrally; single black mark on 4^th^ and 8^th^ intervals	**10**
10	Black marks on central elytra connected by thin lines; most individuals with black transverse band on elytral base (Fig. 13A). Prosternal process slightly narrowed near procoxa (Fig. 13C)	***H.basinotatus***
–	Black mark on central elytra connected by black bands; most individuals without black transverse band on elytral base (Fig. 14A). Prosternal process deeply narrowed near procoxa (Fig. 14C)	***H.kotoshonis***

## ﻿Discussion

In this paper we recognize 13 species belonging to two genera from Japan. Mori (2007) stated that several unrecorded species of the genus *Haliplus* were known from Nansei shoto and other areas of Japan. These unrecorded species are considered to be *H.regimbarti* (recorded by Iwata 2016), *H.angustifrons* (recorded in this paper), and *H.morii* sp. nov. (described in this paper).

## Supplementary Material

XML Treatment for Peltodytes intermedius

XML Treatment for Peltodytes sinensis

XML Treatment for Haliplus (Nipponiplus) japonicus

XML Treatment for Haliplus (Nipponiplus) regimbarti

XML Treatment for Haliplus (Haliplus) kamiyai

XML Treatment for Haliplus (Haliplus) morii

XML Treatment for Haliplus (Haliplus) simplex

XML Treatment for Haliplus (Liaphlus) angustifrons

XML Treatment for Haliplus (Liaphlus) basinotatus

XML Treatment for Haliplus (Liaphlus) kotoshonis

XML Treatment for Haliplus (Liaphlus) eximius

XML Treatment for Haliplus (Liaphlus) ovalis

XML Treatment for Haliplus (Liaphlus) sharpi

## ﻿Additional information

### Author ORCIDs

Masakazu Hayashi https://orcid.org/0000-0003-3708-9005

Tomofumi Iwata https://orcid.org/0000-0002-7738-3199

Hiroyuki Yoshitomi https://orcid.org/0000-0001-8416-9249

### Data availability

All of the data that support the findings of this study are available in the main text or Supplementary Information.

## Supplementary materials

Supplementary material 1 The list of specimens examined in this study Data type table (.xlsx) https://binary.pensoft.net/file/872280This dataset is made available under the Open Database License (http://opendatacommons.org/licenses/odbl/1.0/). The Open Database License (ODbL) is a license agreement intended to allow users to freely share, modify, and use this Dataset while maintaining this same freedom for others, provided that the original source and author(s) are credited.Masakazu Hayashi, Tomofumi Iwata, Hiroyuki Yoshitomi
